# The Road to Recovery: A Two-Year Longitudinal Analysis of Mental Health Among University Students During and After the COVID-19 Pandemic

**DOI:** 10.3390/bs14121146

**Published:** 2024-11-28

**Authors:** Rosie E. Allen, Kevin D. Hochard, Chathurika Kannangara, Jerome Carson

**Affiliations:** 1 School of Psychology, Faculty of Health and Wellbeing, University of Bolton, Deane Road, Bolton BL3 5AB, UK; c.kannangara@bolton.ac.uk (C.K.); j.carson@bolton.ac.uk (J.C.); 2 Division of Psychology, University of Chester, Parkgate Road, Chester CH1 4BJ, UK; k.hochard@chester.ac.uk

**Keywords:** mental health, psychological wellbeing, university students, anxiety, psychological distress, flourishing, higher education, longitudinal research, COVID-19

## Abstract

Longitudinal research into the impact of COVID-19 on university students’ mental health beyond the pandemic is lacking. This study aims to address the gap in the literature by tracking the mental health of university students over a two-year period, spanning the COVID-19 pandemic and its aftermath. A two-year longitudinal study surveyed a sample of university students (*n* = 302) three times between May 2020 and May 2022. Students’ psychological distress, generalised anxiety, flourishing, and personal wellbeing were assessed at each time point. It was found that students’ psychological distress levels spiked in May 2021 (T1) during the first year of the pandemic but reverted back to similar levels seen in May 2020 (T0) at the two-year follow-up (T2). While generalised anxiety gradually improved, both students’ psychological distress and generalised anxiety remained considerably worse than pre-pandemic norms obtained in other studies. Students’ flourishing scores remained very low, while their life satisfaction and state happiness improved slightly between May 2021 (T1) and May 2022 (T2). These findings clearly demonstrate that students’ mental health is still in crisis, even after the COVID-19 pandemic. More needs to be done to support students beyond the pandemic generally, including this particularly unique cohort of students who endured unprecedented challenges for prolonged periods, and who are now transitioning into the working world. Practical implications and recommendations are discussed.

## 1. Introduction

Pandemics have widespread impacts on global health, economies, and societies, and are expected to become more frequent in the future. In order to better understand how recovery can be effectively managed, the latest COVID-19 pandemic can be used as a model to assess strategies and outcomes in the aftermath of such crises.

University students were one of the most vulnerable groups to the consequences of the COVID-19 pandemic [1,2]. Social restrictions meant that students were deprived of their usual social support networks which are crucial for buffering the adversities associated with transitioning into and managing university life [3]. The transition to remote learning and the lack of campus activities meant that students had limited interaction with peers and lost face-to-face contact with lecturers, heightening feelings of loneliness and exacerbating distress [4,5,6,7,8]. Unpredictability and a lack of clarity around assessment formats and academic expectations contributed to increased stress and anxiety [9,10]. These factors have created unique challenges that have significantly affected the mental health of university students, and as a result, research into the mental health impacts of the pandemic on university students was declared a top priority [11]. A substantial body of evidence has demonstrated the severe mental health impacts of the pandemic on university students, with increased stress, anxiety, and depression, along with the exacerbation of pre-existing mental health conditions [12,13,14,15,16,17,18]. For instance, a recent systematic review and meta-analysis [19] showed a pooled proportion of generalised anxiety across 36 studies at a prevalence of 41%, suggesting that students are experiencing high rates of anxiety. Increasing poor mental health generated a significantly higher demand for mental health services [20], which meant many students did not receive timely access to support, and this caused further distress [21,22,23].

The re-introduction of a typical campus environment in the post-pandemic period meant students could reconnect with peers and academic staff, providing crucial physical and emotional support. As academic life stabilised, students began to restore a sense of control and predictability in their lives, which positively impacted their mental health [24,25]. Subsequently, some recent research has shown that the mental health of university students has started to improve [26]. For instance, Lemyre et al. (2024) carried out a longitudinal study of around 150 students across four universities in England, capturing data in April 2021 and November 2021. They found that initial wellbeing was negatively impacted and that there was a significant improvement in students’ wellbeing between April and November 2021 [26].

Nonetheless, longitudinal research on the impact of the COVID-19 pandemic on university student mental health post-pandemic is significantly lacking. There still remains a crucial need to understand the trajectory of mental health impacts, providing a timeline of students’ wellbeing during and after the pandemic. Therefore, more comprehensive research is needed to provide deeper insights into the lasting effects of the pandemic on students’ mental wellbeing. It is important for universities, policymakers and mental health professionals to have a greater understanding of the complexities of students’ mental health trajectories to inform support strategies and address the evolving needs of students. Therefore, the main aim of this study was to address the gap in the literature by assessing the mental health of university students during and after the COVID-19 pandemic. By tracking their mental health over a two-year period—collecting data in May 2020 (T0), May 2021 (T1), and May 2022 (T2)—this study aimed to assess whether there were significant improvements in the wellbeing of university students after the COVID-19 pandemic (T2). Considering the unprecedented nature of the COVID-19 pandemic and its multifaceted impact on university students [12,13,14,15,16,17,18], the authors hypothesised that there would be a significant improvement in the overall mental health of university students in the post-pandemic period across measures of mental illness and of positive mental wellbeing. This would align with what recent studies have found, indicating an improvement in university students’ wellbeing in the later phases, and coming out of the pandemic [24,25,26]. Specifically, it was hypothesised that psychological distress would increase initially (T0 to T1), reflecting heightened stress during the first years of the pandemic, before decreasing by the end of the two-year period (T2) as students adjusted to post-pandemic life. Generalised anxiety was hypothesised to gradually decrease over time (T0 to T2) as uncertainties related to the pandemic diminished and students began returning to normal. Flourishing and wellbeing scores were also hypothesised to show minimal or no improvement by T1, given the ongoing pandemic, with a more noticeable increase by T2 as conditions normalised following the end of the pandemic.

## 2. Materials and Methods

### 2.1. Design

This was a longitudinal study that adopted a repeated measures online survey design, aiming to track the mental health of university students from the early stages of the COVID-19 pandemic to the post-pandemic era. Data were collected online at three time points. First, between 14 and 16 May 2020 (T0), where those completing T0 (*n* = 1578) were invited to complete a follow-up one year later (T1) between 14 May and 4 June 2021 (*n* = 554). Those who completed the one-year follow-up (T1) were invited to complete the survey again another year later (T2), between 10 June and 24 June 2022 (*n* = 302). Data were collected through Prolific, which is an online crowdsourcing platform that was specifically designed for use in a research context [27]. Certain features available to researchers such as pre-screening and ‘allowlisting’ participants permit the collection of longitudinal data [28]. A timeline can be seen in Supplementary Materials, which offers context to the data collection periods in reference to what was happening in the UK for the general public, and university students specifically, around each time the data were collected.

### 2.2. Participants

In order to be deemed eligible to participate, Prolific users needed to be university students, currently residing and studying in the UK. A total of 1578 students completed the survey at T0 (May 2020), 554 completed the survey at T1 (May 2021), and a total of 302 university students in the UK completed the two-year follow-up (T2) survey online (May 2022). The attrition rate after two years was high, at 80.8%, and will be discussed further in the limitations. Table 1 presents the demographic characteristics of the participant sample at T0 (May 2020). At T0, participants were mostly female (76.5%), with a mean age of 24.6. Of the 302 participants at T2, 89 were recent graduates and were no longer studying at university. We asked the 89 recent graduates what they were doing now they had left university. Mostly, they were in full-time employment (*n* = 63) or part-time employment (*n* = 8). Some were unemployed and looking for work (*n* = 9), unemployed but not looking for work (*n* = 4), or self-employed (*n* = 5).

### 2.3. Measures

At each phase of data collection, participants were asked to complete a series of mental health-related standardised measures.

#### 2.3.1. Psychological Distress (CORE-10)

Clinical Outcomes in Routine Evaluation (CORE-10) is a 10-item measure of psychological distress [29]. This scale is rated on a 5-point frequency of occurrence basis (0–4), from “not at all” to “most or all of the time” in response to items such as “I have felt tense or anxious”. Participants are asked to rate their response to the items based on how they have been over the last week. Items 2 and 3 are positively worded and are therefore reverse-coded before a total CORE-10 score is calculated, where a higher score is reflective of higher psychological distress. The reliability and validity of this scale have been extensively tested and confirmed [30,31,32]. Internal reliability analysis using the data from the present study (T0) revealed a Cronbach’s alpha estimate of 0.84.

#### 2.3.2. Generalised Anxiety (GAD-7)

The generalised anxiety disorder (GAD-7) scale is a 7-item measure of generalised anxiety disorder and is often used as a screening tool and symptom severity measure for clinically significant anxiety disorders in outpatient settings [33]. This scale is rated on a 4-point (0–3) frequency of occurrence basis, from “not at all” to “nearly every day”, in response to items such as “Worrying too much about different things”. All items are negatively worded and are added together to calculate a total GAD-7 score. Participants are asked to rate their responses based on how they felt over the past 2 weeks. The GAD-7 scale has been shown to have good reliability and validity [15,34,35]. Internal reliability analysis using the data from the present study (T0) revealed a Cronbach’s alpha estimate of 0.90.

#### 2.3.3. Flourishing (PERMA-Profiler)

The PERMA-Profiler is a 23-item measure of flourishing [36]. Five PERMA subscales denote the overall flourishing score: positive emotions (3 items), engagement (3 items), relationships (3 items), meaning (3 items), and accomplishments (3 items). This scale also includes eight filler items aimed to control response bias that cover physical health (3 items), negative emotions (3 items), loneliness (1 item), and overall happiness (1 item). Participants rate their responses along an 11-point rating scale, which includes positively and negatively worded items. All items, when reverse coded, are scored from 0, which represents “never”, “terrible”, and “not at all”, to 10, which represents “always”, “excellent”, and “completely”, depending on the item. For example, Item 5 asks “How often do you feel joyful?” with anchors of 0 (“Never”) and 10 (“Always”). This scale has good reliability and validity and has been successfully used by other researchers [37,38]. For the overall flourishing score, internal reliability analysis using the data from the present study (T0) revealed a Cronbach’s alpha estimate of 0.93.

#### 2.3.4. Personal Wellbeing (ONS-4)

This is a short, 4-item measure of personal wellbeing, adapted from the ONS Annual Population Survey [39,40]. Each item of the ONS-4 focuses on a specific concept: life satisfaction, worthwhile life, happiness, and anxiety. Two items (Life satisfaction and worthwhile life) are general questions about participants’ feelings on those aspects of their lives. For example, one item focused on life satisfaction asks “Overall, how satisfied are you with your life nowadays?” The other two items (happiness and anxiety) specifically ask participants how happy/anxious they felt yesterday. The ONS-4 is scored in the form of a rating scale from 0 (“Not at all”) to 10 (“Completely”), where each item is considered independently.

### 2.4. Procedure

The survey was compiled on Qualtrics and uploaded to Prolific, an online platform designed to recruit participants for online research. Participants were invited at T0 if they were studying at university at the time. Participation in this research was voluntary, and after participants read the study information and gave written informed consent to take part, they were asked to provide basic demographic information including age and gender. Participants were then asked to complete a series of questionnaires: CORE-10, PERMA-Profiler, GAD-7, and ONS-4. Participants were paid GBP 1.25 for each time they completed the survey, taking approximately 10 min. Participants who were lost to follow-up phases of the research were traced and contacted once via Prolific to offer the study information and an invitation to take part [41]. Ethical approval for the study was obtained from the University of Bolton Ethics Committee in line with British Psychological Society guidelines.

### 2.5. Statistical Analysis

In this research, we used the platform Prolific, which provides each participant with a unique and consistent participant ID. This ID remains unchanged each time a participant completes a survey on the platform, making it possible to track responses over multiple time points. By using this consistent identifier, we were able to accurately match participants’ responses across all time points, ensuring reliable longitudinal data-pairing for our analysis. Sum scores were computed for the CORE-10, GAD-7, and PERMA measures with mean imputation for up to 10% missing data per instrument [42]. The repeated measures outcomes were restructured from a wide format to a long format and followed the nested structure whereby each participant (*n* = 302) provided data for 3 time points. All analyses were performed using the open software Jamovi (version 2.1.3) [43].

Mixed-effect models accounting for fixed and random effects were performed in the General Analyses for Linear Models in Jamovi (GAMLj), package version 2.4.8 [44]. These analyses accounted for both the overall trends over time and the individual variations.

Seven models (one per outcome) were computed, which included the repeated measure (T0, T1, and T2) as our fixed-factor predictor (time) and included correlated participant-level random intercepts, using a restricted maximum-likelihood estimate (REML) with the bobyqa optimizer, as this function produces less biased estimates of the variance components and is widely employed in Generalised Linear Mixed Models (GLMMs) [45].

Little’s missing completely at random (MCAR) test, with Expectation Maximisation, was used to assess whether missing data on the outcome variables were missing completely at random (MCAR) [46]. Further to this, a chi-squared test was performed to determine gender differences in dropout, and an independent samples Kruskal–Wallis test was carried out to look for age differences in dropout. Finally, independent samples t-tests were conducted to determine whether mental health at T0 differed for those who dropped out compared to those who were retained.

## 3. Results

Means and standard deviations for study variables across each of the time points are reported in Table 2. Mixed-effect models for flourishing, distress, and generalised anxiety are reported in Table A1 while ONS outcomes of life satisfaction, life worthwhile, happiness, and anxiety are reported in Table A2.

The mixed-effect models reported displayed approximately normally distributed, homoscedastic residuals. For all models, a small marginal R^2^ relative to the condition R^2^ suggests a large impact of individual differences and a small effect of the fixed factor. The ICC for the models showed that the clustering for participants was substantial.

With respect to flourishing, we observed a small yet significant negative effect of time, with reductions from T0 to T1 and from T0 to T2. Distress increased significantly from T0 to T1 but was not significantly different from T0 to T2. Generalised anxiety at T1 did not significantly differ from T0 but did significantly reduce from T0 to T2. Similarly, both life satisfaction and happiness displayed no difference from T0 to T1 but increased from T0 to T2. Life worthwhile did not demonstrate any significant change over time from T0. However, anxiety demonstrated significant reductions from T0 to T1 and T2.

### Retention and Dropout

At T0 (May 2020), there were 1578 responses. At the two-year (T2) follow-up (May 2022), there were 302 responses. Overall retention after 2 years was 80.8%.

A chi-squared test revealed that females were more likely to be retained, and males were more likely to drop out, χ^2^(1, *n* = 302) = 299, *p* < 0.001 As age was not normally distributed, an independent samples Kruskal–Wallis test was carried out to test whether the age of participants at T0 was associated with dropout from the 2-year follow-up. The findings showed that age was significantly associated with retention, *H*(2002) = 6.45, *p* < 0.05, where older students at T0 were more likely to be retained at T2.

However, independent samples t-tests were conducted to determine whether mental health outcomes at T0 (May 2020) differed for those who dropped out compared to those who were retained at T2 (May 2022). The findings indicated that there were no significant differences at T0 between participants who stayed for the entirety of the research (*n* = 302) and participants who dropped out (*n* = 1276) for psychological distress (*p* = 0.603), anxiety (*p* = 0.789), flourishing (*p* = 0.506), life satisfaction (*p* = 0.302), worthwhileness (*p* = 0.551), happiness felt yesterday (*p* = 0.637), or anxiety felt yesterday (*p* = 0.321). These findings suggest that those who stayed in the research and those who dropped out were similar in terms of mental wellbeing at T0 (May 2020). Therefore, females and older participants were more likely to be retained. However, retention was not related to the mental health of participants at T0.

This pattern is supported by Little’s missing completely at random (MCAR) test conducted using Expectation Maximisation on our outcome variables, including CORE-10, PERMA-Profiler, GAD-7, and four ONS items (χ^2^(343) = 255.55, *p* = 1.000), which indicated that the data were missing completely at random (MCAR). Therefore, no further adjustments for missing data are necessary.

## 4. Discussion

The findings indicate that psychological distress levels increased between May 2020 and May 2021, likely due to the continuous effects of lockdowns, academic disruptions, and uncertainty [47,48]. The increase in students’ psychological distress scores in May 2021 (T1) could have been impacted based on the nature of the questioning in the CORE-10 measure, which asks participants to rate their responses based on their feelings over the last week. For instance, at this time, in-person lectures had recently been re-introduced with periodic testing and mandatory mask-wearing (see Supplementary Materials), which could have heightened levels of anxiety related to their personal health and changing academic routines. It is also important to note that in the UK, May generally tends to be a period in the lead-up to exams at university, and so this could be a contributing factor, as students adjust to evolving forms of assessment in the changing climate. By the second year (May 2022), psychological distress levels decreased, returning to similar levels to that at T0 (May 2020), aligning with recent research in a non-student sample [49]. This might be due to the fact that two years into the pandemic, these cumulative effects, particularly around fear and uncertainty, had started to diminish [50], and people had adapted to changing academic and living conditions [51]. It is also possible that greater familiarity with pandemic-related changes, and reduced perceived risks following public health measures, such as the vaccination rollout, might have encouraged a sense of control in people’s lives and contributed towards the reduction in distress in May 2022 (T2) [52]. At this time, all legal restrictions, including the requirement to wear a mask, had recently been lifted (see Supplementary Materials) and may have resulted in a reduction in their levels of psychological distress.

Current findings also revealed that generalised anxiety levels remained stable during the first year (T0–T1), but that they started to improve by May 2022 (T2), gaining support from recent research [53]. Similarly, anxiety yesterday (as measured by ONS4) demonstrated significant reductions from T0 to T1 and further to T2, indicating that both state and trait levels of anxiety were improved by May 2022 (T2). Anxiety levels were highest in May 2020, at a time when the COVID-19 pandemic was in its early stages with greater levels of fear and uncertainty [54,55]. As time went on, it is likely that students were becoming more familiar with the new living conditions, academic adjustments, and social restrictions, leading to a gradual reduction in their generalised anxiety levels [53,56], as shown by the reductions in students’ scores at T1 and T2. Specifically, during May 2021, students, like the general population, were undergoing a period of relaxing of social restrictions, where the nation was at Step 3 of the roadmap that was devised by the British government to bring us out of lockdown indefinitely. Students during this period were allowed to gather in groups of six indoors and fifty outdoors, and indoor venues such as pubs and cinemas had recently re-opened which may have helped to facilitate leisure and social activities and alleviate some anxiety (see Supplementary Materials). By May 2022, students’ social and academic environments had returned to a typical pre-pandemic experience as all legal restrictions had been lifted a few months prior (see Supplementary Materials). After such long periods in which this was restricted, increasing possibilities for social interaction were likely to contribute towards reduced anxiety in university students, due to the strong association between positive social contact and decreased anxiety [57,58]. This coincides with Relational Regulation Theory [59] which posits that an individual’s relationships with other people can help to regulate their emotions and help them cope with stressful events.

Nevertheless, even in May 2022 (T2), when we saw some significant improvements, students’ generalised anxiety remained significantly higher than scores that were captured by other researchers (*M* = 4.75) before the COVID-19 pandemic [60]. On top of this, their psychological distress was still around 3x higher than normative scores of 4.7 that were captured by other researchers before the pandemic [29], reinforcing the long-lasting impact of the pandemic on students’ mental health [61,62]. It is likely that the prolonged stress from the pandemic, including academic and social challenges, financial instability, and uncertainty about the future, were collectively contributing towards sustained psychological distress and anxiety in students, even post-COVID-19, as reflected in sustained psychological distress at T2. Further, a study carried out by King’s College London using the data collected from the Student Academic Experiences Survey revealed that over 15% of UK students were still reporting a mental health problem in 2023 [62].

The findings demonstrate that students’ flourishing decreased over the two-year period, likely reflecting the prolonged stress and disruption caused by the pandemic [63]. More specifically, we know that students’ distress and anxiety remained elevated in this post-pandemic era when drawing comparisons to normative scores from studies that were carried out pre-pandemic [29,60]. These prolonged mental health problems are likely hindering students’ abilities to experience positive emotions. Dealing with academic adjustments, ongoing uncertainty, and poor mental health is likely to impact students’ ability to engage in their usual academic and social activities [64]. At a point when students and recent graduates may have been struggling with re-transitioning to typical social and academic life, including a reported rise in social anxiety post-pandemic [65], this may negatively impact students’ relationships and social fulfilment. Finally, not only did students face cancelled and delayed graduation ceremonies, but the prominent lack of practical learning opportunities during the pandemic [66] may negatively impact their skills and competence [67], which is likely to affect their sense of accomplishment [11]. Therefore, it is plausible that students have been unable to meet the criteria and demands to achieve flourishing mental health [68], as students’ ability to experience positive emotions, engagement, relationships, meaning in life, and accomplishments may have been severely limited, if not withheld entirely, during the COVID-19 pandemic. It is important to note, however, that flourishing scores remained particularly low even after the COVID-19 pandemic when students were more likely to have regained access to their preferred academic and social routines and support systems. This could potentially highlight the lasting impact of having sustained restrictions and access to the things that bring them joy, security, and support. Additionally, as a consequence of the pandemic, students may re-evaluate their priorities and feel a sense of uncertainty about the future and their career prospects [1,47], which could influence their perceptions of meaning in their lives.

While both life satisfaction and happiness yesterday showed no difference between T0 and T1, both had improved by T2. Interestingly, students’ scores of happiness yesterday appeared higher (*M* = 6.27) in May 2022 (T2) than the pre-pandemic norm of 5.5 as captured by the Office for National Statistics (ONS) in 2018 [40]. As a state measure of happiness, that is, capturing students’ immediate emotional states at a specific moment in time [69,70], their happiness felt yesterday at the time of data collection in May 2022 may have been particularly sensitive to environmental and societal changes occurring. For instance, the lifting of legal restrictions and the return to normal social and university life may have boosted their immediate emotional wellbeing, resulting in higher happiness yesterday scores.

Current findings revealed that life worthwhileness did not demonstrate any significant change over time from T0 (May 2020) to T2 (May 2022). The psychological construct of life being worthwhile may, naturally, be more stable over time and less sensitive to temporary changes or external challenges [71]. While other measures, such as happiness yesterday and anxiety yesterday, demonstrated significant changes over the duration of the pandemic, the fundamental aspects of what makes life worthwhile for students may have remained stable. For instance, despite the transition to distance learning, continuity of learning meant that students could still pursue their ongoing academic and personal goals, as their long-term goals of academic success and career progress were likely to remain constant.

### 4.1. Limitations

Self-report measures were used, which are often confounded with social desirability [72], to which individuals of certain demographic or cultural backgrounds are more likely to provide socially desirable answers [73]. Nevertheless, psychological research is dominated by self-report measures, and standardised measures were used that have had extensive reliability and validity testing. Also, a large proportion of the research samples were female. However, this was expected due to the unequal gender balance apparent in higher education [74] and the increased likelihood of females participating in online research [75]. The length of longitudinal research is associated with higher dropout rates [76], and ‘attrition bias’ refers to the systematic differences between those who are retained and those who drop out of research [77]. In the current study, the attrition rate after two years was 80.8%, which is out of the range of the commonly reported (30–70%) attrition rate in longitudinal epidemiological studies [78]. This could potentially be related to increased binge drinking [79,80], increased job loss and employment instability [8], and reduced access to typical social support systems [81] that were reported during the COVID-19 pandemic, which are all associated with higher dropout rates [79,82]. Additionally, previous research has shown that mental health problems predict non-response in longitudinal research [83], especially in university students [84]. Indeed, high levels of psychological distress [80,85], anxiety, and depression [86] can predict attrition. In the current study, psychological distress and generalised anxiety scores were considerably high across all time points and were shown to be worse than the pre-pandemic norm, so attrition was likely to be impacted. In this study, females and older students were more likely to be retained, which may influence the generalisability of findings. This research is limited to drawing inferences and conclusions from data captured during and after the COVID-19 pandemic (May 2020–May 2022) and does not allow for pre-pandemic comparisons with this sample.

### 4.2. Practical Implications and Recommendations

After more than four years since the beginning of the pandemic, it is crucial to understand how the mental wellbeing of university students fluctuated throughout the pandemic, and how they are faring in a post-pandemic context. We have only recently started to understand the long-term psychological impacts of COVID-19 on university students. The current longitudinal study provided the ability to provide a picture of university students’ mental health throughout an unprecedented global health pandemic. These findings show that students are still particularly vulnerable and need continued support extending beyond the pandemic. These findings can provide several practical implications and recommendations for further research, with regard to addressing the mental health of university students post-pandemic. While there have been some signs of improvement, students’ psychological distress and generalised anxiety are still elevated and remain considerably worse than pre-pandemic norms. Also, student flourishing continued to gradually decline over the course of the pandemic, remaining low even after personal and academic life began to return to that of a pre-pandemic state.

#### Policy Implications

Clearly, universities need to adapt and improve the mental health services they have available to students to help them cope with the lasting impacts. The university setting plays an important role in shaping the future of its students and is the perfect platform for health and mental health promotion. Further investments are needed to screen and monitor students’ health and wellbeing to help with the identification of students who could benefit from health and wellbeing services. More inventive ways of supporting and protecting students’ wellbeing are needed moving forward to redefine mental health services, including rethinking how policies can support this. For example, greater efforts to consult with students themselves to co-produce in-person and digital programmes that could complement the curriculum, improve engagement with and the relevance of services, and alleviate the strain on overwhelmed mental health services.

Policymakers should consider the effectiveness of Positive Psychology Interventions (PPIs) in mitigating mental health problems and protecting university students’ wellbeing, whereby such approaches are integrated into mental health support services. Worsley et al. (2022) recently conducted a systematic literature review of other reviews, focusing on interventions designed to support the mental health and wellbeing of university students. The authors reviewed 27 reviews and concluded that the strongest or most effective interventions to improve student mental health were mindfulness-based interventions and cognitive–behavioural interventions [87]. Future research should continue to evaluate the effectiveness of different mental health interventions in this context, to help refine and adapt existing interventions, as well as develop new evidence-based practices that are specifically targeted to the needs of this unique group.

This is a cohort of students who had particularly unique personal and academic experiences, which are not only important to consider during the pandemic, but these impacts are likely to continue to be felt long after the pandemic. After all, nearly a third of the current sample at the two-year follow-up were recent graduates, and they too were still experiencing considerably poor mental health. These recent graduates did not have the experiences that past generations of students would have had, and this will have impacted their competence and confidence in being successful in securing graduate employment [88]. Therefore, we need to consider the longer-term impacts beyond university, advocating for continued graduate support and further research into how their experiences during that time might have impacted their life, personally, but also professionally, after university.

## 5. Conclusions

Improvements in psychological distress, both state and trait anxiety, and overall personal wellbeing suggest a positive trend towards mental health recovery post-pandemic in this unique cohort of university students. However, mental health scores are still poor, remaining considerably worse than the pre-pandemic norm. These findings demonstrate the desperate need for continued attention in addressing students’ mental health challenges beyond the pandemic. The need for more mental health support for university students remains the same now as it was during the COVID-19 pandemic. It also calls for continued support for this disproportionately impacted cohort in the post-pandemic era as they transition into the graduate job market. The current findings will contribute towards a greater awareness of the short- and long-term psychological impacts on student mental health, helping to inform and shape future general mental health support strategies, as well as specifically reducing risks in the event of a future global health pandemic.

## Figures and Tables

**Table 1:** Demographic characteristics of the sample in May 2020 (T0).

Demographic Characteristic		Number of Participants (*n*)	Percentage of Sample (%)
Gender	Female	231	76.5
	Male	69	22.8
	Non-Binary	2	0.7
Age	18–19	62	20.6
	20–21	100	33.1
	22–24	49	16.2
	25–30	37	12.3
	31–40	35	11.7
	41+	19	6.1
Year of Study	Foundation	44	14.6
	First Year	92	30.5
	Second Year	71	23.5
	Third Year	50	16.6
	Fourth Year	33	10.9
	Masters	10	3.3
	PhD	2	0.6

**Table 2:** Mean and standard deviation of UK students at each time point for psychological distress, generalised anxiety, flourishing, and personal wellbeing.

	T0 (*n* = 302)		T1 (*n* = 297)		T2 (*n* = 299)	
	Mean	SD	Mean	SD	Mean	SD
**Flourishing**	95.60	23.73	92.29	26.37	92.96	27.03
**Distress**	13.69	7.26	15.30	5.64	13.76	5.72
**Generalised Anxiety**	7.44	5.30	7.06	5.03	6.25	5.06
**Personal Wellbeing**						
Life Satisfaction	5.76	2.34	5.96	2.30	6.04	2.38
Life Worthwhile	6.05	2.44	6.00	2.44	6.16	2.45
Happiness Yesterday	5.81	2.51	6.05	2.48	6.27	2.44
Anxiety Yesterday	5.56	2.95	4.44	2.96	3.89	2.87

## Data Availability

The original data presented in the study are openly available in FigShare at https://doi.org/10.6084/m9.figshare.26262254 (accessed on 24 November 2024).

## Supplementary Materials

The following supporting information can be downloaded at https://www.mdpi.com/article/10.3390/bs14121146/s1: Figure S1: COVID-19 Timeline in the UK: Context for Data Collection.

## Appendix A

**Table A1:** Mixed-effect models of time on flourishing, psychological distress, and generalised anxiety.

**Fixed Effects**	**Flourishing (PERMA)**					**Distress (CORE-10)**					**Generalised Anxiety (GAD** **-** **7)**				
		**95% CI**					**95% CI**					**95% CI**			
	**Est**	**LL**	**UL**	***t***	***p***	**Est**	**LL**	**UL**	***t***	***p***	**Est**	**LL**	**UL**	***t***	***p***
Intercept	93.59	90.99	96.20	70.44	<0.001	14.24	13.66	14.83	47.79	<0.001	6.92	6.42	7.42	27.34	<0.001
T1–T0	−3.34	−5.62	−1.07	−2.88	0.004	1.62	0.93	2.32	4.57	<0.001	−0.37	−0.90	0.15	−1.39	0.164
T2–T0	−2.69	−4.96	−0.41	−2.32	0.021	0.11	−0.59	0.80	0.30	0.764	−1.19	−1.71	−0.66	−4.44	<0.001
**Random Effects (Intercept)**	**σ^2^**	**ICC**	**Model Fit**			**σ^2^**	**ICC**	**Model Fit**			**σ^2^**	**ICC**	**Model Fit**		
			**AIC**	**R^2^ marginal**	**R^2^ conditional**			**AIC**	**R^2^ marginal**	**R^2^ conditional**			**AIC**	**R^2^ marginal**	**R^2^** **conditional**
Participant	462.24	0.70	7919.90	<0.01	0.70	20.34	0.52	5576.88	0.01	0.53	15.64	0.59	5174.96	0.01	0.60

Note: Est: Estimates; LL: Lower Limit; UL: Upper Limit; σ^2^: residual variance; ICC: intraclass correlation coefficient; AIC: Akaike information criterion; R^2^ marginal: variance explained by the fixed effects over the total (expected) variance of the dependent variable; R^2^ conditional: variance explained by the fixed and random effects over the total (expected) variance of the dependent variable.

**Table A2:** Mixed-effect models of time on personal wellbeing (ONS).

**Fixed Effects**	**Life Satisfaction**					**Life Worthwhile**					**Happiness**					**Anxiety**				
		**95% CI**					**95% CI**					**95% CI**					**95% CI**			
	**Est**	**LL**	**UL**	***t***	***p***	**Est**	**LL**	**UL**	***t***	***p***	**Est**	**LL**	**UL**	***t***	***p***	**Est**	**LL**	**UL**	***t***	***p***
Intercept	5.92	5.69	6.14	51.13	<0.001	6.07	5.83	6.31	49.23	<0.001	6.04	5.82	6.26	54.03	<0.001	4.63	4.44	4.82	47.31	<0.001
T1–T0	0.19	−0.05	0.43	1.59	0.113	−0.06	−0.29	0.18	−0.48	0.629	0.23	−0.07	0.54	1.49	0.136	−1.12	−1.59	−0.65	−4.66	<0.001
T2–T0	0.27	0.03	0.51	2.21	0.028	0.10	−0.14	0.33	0.83	0.409	0.46	0.15	0.76	2.96	0.003	−1.67	−2.14	−1.20	−6.97	<0.001
**Random Effects (Intercept)**	**σ^2^**	**ICC**	**Model Fit**			**σ^2^**	**ICC**	**Model Fit**			**σ^2^**	**ICC**	**Model Fit**			**σ^2^**	**ICC**	**Model Fit**		
			**AIC**	**R^2^ marginal**	**R^2^ conditional**			**AIC**	**R^2^ marginal**	**R^2^ conditional**			**AIC**	**R^2^ marginal**	**R^2^ conditional**			**AIC**	**R^2^ marginal**	**R^2^ conditional**
Participant	3.27	0.60	3765.46	0.00	0.60	3.84	0.64	3778.08	0.00	0.64	2.55	0.42	4031.51	0.01	0.42	0.00	0.00	4470.42	0.05	0.05

Note: Est: Estimates; LL: Lower Limit; UL: Upper Limit; σ^2^: residual variance; ICC: intraclass correlation coefficient; AIC: Akaike information criterion; R^2^ marginal: variance explained by the fixed effects over the total (expected) variance of the dependent variable; R^2^ conditional: variance explained by the fixed and random effects over the total (expected) variance of the dependent variable.
