# Supplementary material for: The Road to Recovery: A Two-Year Longitudinal Analysis of Mental Health Among University Students During and After the COVID-19 Pandemic
Source: Behav Sci (Basel). 2024 Nov 28;14(12):1146. doi: 10.3390/bs14121146 (PMC11673234; doi:10.3390/bs14121146)
Supplement: Supplementary file 1 [file behavsci-14-01146-s001.zip › behavsci-3262268-supplementary.pdf]

The Road to Recovery: A Two-Year Longitudinal Analysis of Mental Health Among University Students During and After the Covid-19 Pandemic

Covid-19 Timeline in the UK: Context for Data Collection

11th March 2020

World Health Organisation declared the Covid-19 outbreak a global health pandemic

Strict lockdown restrictions are announced in a televised address by the Prime Minister, ordering people to "stay at home"

23rd March 2020

University campuses were closed, all face-to-face teaching was suspended and all forms of teaching, learning and assessment were moved online. All exams were cancelled and scheduled graduation ceremonies were postponed

Queen Elizabeth II made a televised broadcast to the British public, the same day that the Prime Minister Boris Johnson was admitted to hospital after testing positive for the virus.

5th April 2020

16th April 2020

Lockdown restrictions were extended for a further three weeks

5th May 2020

The UK had the second highest daily death toll in the world

12th - 14th May 2020

***n*=1578 university students' from the UK completed the online survey, to capture their mental health in the early stages of the pandemic.**

T0

4th July 2020

Gradual easement out of the first national lockdown saw the reopening of pubs, restaurants and hairdressers.

May - June 2020

Lockdown was easing slowly across the country. By the 1st of June, schools were taking a phased approach to re-opening and two weeks later, non-essential shops were reopened in England. On the 23rd of June, the Prime Minister announced the relaxation of nationwide Covid-19 restrictions and a two metre social distancing rule was enforced.

July 2020

August 2020

Lockdown restrictions continued to ease, with the re-opening of some indoor venues

Sep 2020

New rules were introduced including the 'Rule of Six' and a 10pm curfew.

The academic year of 2020/2021 commenced with most universities favouring a blended learning approach.

Covid-19 cases were on the rise which resulted in many universities around the UK placing students into isolation, confined to their accommodation for two weeks. Protests and petitions took place as isolated students shared their frustrations

5th November 2020

A second lockdown in England was enforced for four weeks.

2nd + 8th December 2020

Lockdown ends and England entered into a three-tier system of restrictions. Prime Minister announces tough rules over the Christmas period. On the 8th, vaccinations began.

Dec 2020

21st December 2020

Tier 4 restrictions were enforced in several areas of England.

6th Jan 2021

England entered into a third national lockdown which closely coincided with the commencement of the second term for university students.

22nd February 2021

The British Government published a roadmap for lifting lockdown restrictions in the UK

March 2021

Step 1 of the roadmap was initiated that allowed outdoor gatherings of either six people or two households. Outdoor sports facilities re-opened and the 'stay at home' order ended.

8th March 2021

Primary and secondary school re-open, but those in higher education generally continued to learn remotely or progressed into blended learning.

April 2021

Step 2 of the roadmap commenced that saw non-essential retail, public buildings and outdoor venues such as theme parks and zoos re-open. However, mixing indoors between different households was still not allowed. General public can access free Covid-19 test kits.

May 2021

Step 3 of the roadmap began which increased the limit of people allowed to mix outdoors to 50 and the 'Rule of Six' came back into force. Indoor venues such as pubs and cinemas re-opened.

17th May 2021

All students allowed to return to in-person lectures (with periodic testing), just before exam period starts.

14th May - 4th June 2021

***n*= 554 students' completed the survey for a 2nd time, one year after they initially completed it.**

T1

14th June 2021

By June 2021, all adults above the age of 18 were able to get their first dose of the vaccine.

Step 4 of the roadmap was delayed by four weeks due to accelerated vaccination programme. Meanwhile, all restrictions on weddings and funerals were removed.

19th July 2021

Step 4 of the roadmap launched, removing the majority of social contact restrictions.

December 2021

British government initiates 'Plan B' in response to the discovery and rapid spread of the Omicron variant. 'Plan B' meant face masks were compulsory in public indoor venues and a NHS Covid Pass became mandatory for certain settings.

Most lectures will be in-person for the 2022/23 academic period. Although, some continued with online and recorded lectures even if practical workshops and seminars are face-to-face.
Students attending in-person lectures must wear face masks.

Jan 2022

Covid-19 cases on the rise following Christmas and New Year celebrations. 'Plan B' extended for three weeks. Fully vaccinated people don't need to take a pre-departure test.

10th - 24th June 2022

***n*=302 students' completed the survey for the final time, marking a two year follow-up.**

T2

February 2022

All legal restrictions are lifted, including requirement to wear face masks.

April 2022

Hospitals remain under enormous strain and PCR tests are no longer free to the general public.
